# Rational suicide: The paradigm of survival

**DOI:** 10.1192/j.eurpsy.2021.467

**Published:** 2021-08-13

**Authors:** S. Torres

**Affiliations:** Psychiatry And Mental Health Department, Centro Hospitalar Barreiro-Montijo, Barreiro, Portugal

**Keywords:** rational suicide, assisted death, suicide, phenomenology

## Abstract

**Introduction:**

Suicide is an intriguing act of the human being. The reasons behind the violation of an instinct for survival is far from being understood. Besides, the emergence of assisted dying is raising even more questions about the concept of rational suicide, defined as a well-thought-out decision to die by whom is mentally competent.

**Objectives:**

Understand the concept of rational suicide, in parallel with suicide, by exploring the views on this debate over the years and elucidating the relationship with mental disorders, mental capacity and patient’s rights.

**Methods:**

Literature review performed on PubMed and Google Scholar databases, using the keywords “rational suicide”, “assisted death”, “suicide”, “phenomenology”, “mental capacity” and “responsibility for life”.

**Results:**

The theological condemnations of suicide – as sin or crime – were put aside with psychiatric development in the last century. Durkheim was the first important precursor of the contemporary view - suicide is a form of mental illness (psychosis or depression) not compatible with rational deliberation. With the increasingly open debate on assisted dying, this vision is being tested by cases of terminally ill patients subjected to experiences that many wouldn’t choose to tolerate. Moral right to self-determination and needless suffering are examples of arguments in favor of rational suicide.

**Conclusions:**

The need for an open discussion about rational suicide is raising, specifically in relation to psychiatric disorders, mainly to resolve the conflict between the duty of care of psychiatrists and the autonomy of patients.

**Disclosure:**

No significant relationships.

